# Developing Trans-Affirming Health Services in an Underserved Area: An Intersectional Approach

**DOI:** 10.1089/trgh.2018.0011

**Published:** 2018-07-01

**Authors:** Oralia Loza, Paulina Hernandez, Jessica Calderon-Mora, Shaked Laks, Marie Leiner, Sireesha Reddy, Patricia Lara, Hector Granados

**Affiliations:** ^1^ Department of Public Health Sciences, College of Health Sciences, The University of Texas at El Paso, El Paso, Texas.; ^2^ Department of Pediatrics, Texas Tech University Health Sciences Center, El Paso, El Paso, Texas.; ^3^ Department of Biomedical Sciences, Department of Family and Community Medicine, Texas Tech University Health Sciences Center El Paso, El Paso, Texas.; ^4^ Department of Radiology, Texas Tech University Health Sciences Center, El Paso, El Paso, Texas.; ^5^ Department of Obstetrics and Gynecology, Texas Tech University Health Sciences Center, El Paso, El Paso, Texas.; ^6^ Department of Speech-Language Pathology, College of Health Sciences, The University of Texas at El Paso, El Paso, Texas.

**Keywords:** barriers to healthcare, Hispanic population, medically underserved populations, transgender healthcare, U.S.-Mexico border

## Abstract

**Purpose:** Gender-nonconforming patients are at higher risk for medical problems that require prompt medical and mental health intervention. Barriers to healthcare for transgender individuals have been well characterized in the literature, but not in low resource settings. The purpose of this paper is to present the barriers encountered when bringing healthcare to transgender children, adolescents, and adults in a medically underserved, predominantly Hispanic area of the United States.

**Methods:** In this medically underserved area on the U.S.-Mexico border, there is a severe shortage of medical expertise for transgender individuals at both the primary- and specialty-care levels. Further, given the mainly Hispanic population, there is an additional culturally based barrier to obtaining medical care for transgender patients.

**Results:** It is important for academic centers in these regions to collaborate to overcome these barriers through a multidisciplinary approach that includes providing education for medical students and physicians in training and identifying medical providers who are able and willing to provide transgender-competent care adapted to local culture and gender norms.

**Conclusion:** In this manuscript, we will describe the efforts of various groups to address the needs of the transgender community in the region.

## Introduction

Health disparities and barriers to healthcare for transgender individuals have been identified at the U.S. national level.^1^ The specific factors that interfere with the delivery of care by physicians are not clear, however, as most of the research that evaluates disparities is performed from the perspective of the patient. Physician knowledge, attitudes, and barriers have been identified for lesbian, gay, bisexual, and transgender (LGBT) care in general, but they have not been addressed for transgender care specifically.^2^

The issues begin in medical and clinical education, as the curricula in most schools do not include transgender care and do not provide the requisite knowledge to ensure that future doctors are confident and competent to provide quality care to these individuals.^3^ In addition, current efforts to include transgender care in the curriculum of medical schools are focused on physicians' need for transgender education without an understanding of the non-physician-related barriers to providing care.^4^ These barriers may be based on ethnicity, religion, culture, economic class, race, education, age, mental health condition, or lack of insurance as well as related issues. Although some of these barriers are experienced by other minority groups, many are unique to or are magnified in the transgender population.^5^ Societal stigma and discrimination are major factors that act as barriers to general healthcare and can lead to further disparities, such as late diagnosis, delayed treatment, and poor health outcomes.^6^

The role of the primary care physicians (PCPs) goes beyond providing medical care to their patients. PCPs are entrusted with an individual's most crucial health and social needs in an intimate setting and are thus afforded an opportunity to eliminate health disparities through training in transgender-specific health needs.^7^ The ability of patients to feel comfortable, be open and honest about their health, and disclose their sexual orientation and gender identity allow for optimum care. Fears of transphobic reactions from providers, concerns about confidentiality and stigma, discrimination from PCPs, and past negative experiences with providers, however, are major reasons why transgender persons have difficulty disclosing to their providers.^8^ As a result, transgender patients who seek healthcare and/or social services may receive insufficient or inappropriate care due to their fear of being stigmatized by their provider. Providers who do not know the sexual orientation or gender identity of their patients, as patients can be reluctant to disclose, may inadvertently incorrectly diagnose, treat, or refer their patients for unnecessary services.^9^

### Transgender healthcare on the U.S.–Mexico border

Transgender healthcare among Hispanics and on the U.S.–Mexico border has been minimally addressed in the literature. A 2011 qualitative Transgender Women's Identity, Sexuality, and Transition (TWIST) Study among 13 transgender women in El Paso, Texas, a U.S.–Mexico border city, 7 of whom were of Mexican descent, found that participants could not identify a local healthcare or social services provider whom they felt was trans-friendly or competent.^10^ Those who had sought services disclosed that they had negative experiences, with the exception of those who had access to health services at the Veterans Affairs hospital. Lack of knowledge among the provider workforce in offering transgender medical care; fear of being stigmatized; lack of clinic structure, forms, and electronic medical records; and lack of insurance coverage were identified as barriers by transgender individuals. In addition, gaps in knowledge and bias among support staff were identified as initial barriers to providing services to the transgender population in our academic institution.

### Cultural needs assessment

In 2012, the City of El Paso Department of Public Health HIV Prevention Program conducted a needs assessment for the Paso del Norte region to evaluate (1) HIV-related attitudes among men who have sex with men (MSM) and transgender women, and (2) cultural factors as a means to improve the program's outreach efforts.^11,12^ Four focus groups were conducted between November and December 2012 and included MSM ages 18–29, MSM 30 years and over, MSM 18–65 years who were Spanish-speaking only, and transgender women ages 18–65 years. With regard to health-related topics among these populations, societal stigma was identified as a barrier to accessing healthcare and social services among MSM and transgender women. Many focus group participants described hardships in their coming-out process and difficulty gaining acceptance from family and social networks due to religious upbringing and “machismo” attitudes.^12^ In some cases, such hardships led to mental health issues, such as depression, and dependence on alcohol and drugs to cope with such reactions. The use of drugs and alcohol was universal among TransLatinas. High-risk sexual behavior, including multiple anonymous sex partners and unprotected sex, also were common.

### Hispanic population

The El Paso County area, a border town with a predominantly Hispanic population (82.2%),^13^ faces unique circumstances, from a cultural and a medical standpoint, as an underserved area. El Paso, Texas is across the U.S.-Mexico border from Cd. Juárez, a predominantly binational, bicultural, and bilingual community,^14^ which forms the largest international metroplex in the world with a population of close to 2 million.^15,16^

In general, Hispanics in the United States face unique barriers to healthcare that lead to greater health disparities. Language, immigration status, cultural persuasion, and lack of health insurance are precursors to poorer health among Hispanics compared with the general population in the United States.^17^ These health disparities lead to health inequalities that result in Hispanics being classified as an underserved population.^18^ Hispanic Americans have attitudes toward transgender communities that are similar to those of the general population. Results of a Pew Research Center^19^ survey regarding support for same-sex marriage indicated that the rate among Hispanics in the United States (46%) is similar to that of the U.S. general public (52%). Fewer than half of 2000 Latino youth in the United States, for whom family acceptance is a primary concern, however, reported a supportive adult to turn to and were twice as likely to feel as though they did not fit into their current communities.^20,21^ Furthermore, acceptance of transgender communities within Hispanic populations varies by such factors as national origin, degree of religious affiliation, and political party association.^22^

### Medically underserved area

In addition to being a culturally underserved area, based on the area having a large Hispanic population, the southwest portion of Texas is considered a partially medically underserved area (MUA), according to the Health Resources and Services Administration (HRSA).^23^ The designation is based on four variables: physicians-to-population ratio, infant mortality rate, population below poverty level, and population over 65 years of age.^23^ The El Paso County area has been designated an MUA since 1994, despite its being considered a rational area to provide medical care services.^24^ The El Paso County area is designated as an MUA mainly because there is a physician in primary care ratio of 116 per 100,000 residents, 58% below what the area needs. Moreover, the area is also short on specialty physicians.^25^ It is considered an area with unusually high needs for primary care, which some attribute to the fact that there are a number of nonresident patients who seek medical attention. Having a population below the poverty line also contributes to this designation. In 2013, the percentage of El Paso County residents with an income below the poverty level was 28.6%, which is above the federal poverty level but below the state poverty level.^26^

### Multidisciplinary transgender healthcare group development

Current guidelines for the care of transgender individuals recommend pretreatment screening and appropriate, regular medical monitoring.^27,28^ The standard monitoring plan for individuals includes the use of PCPs, including, but not limited to, pediatricians, internists, gynecologists, fertility specialists, urologists, surgeons, psychologists, psychiatrists, counselors, social service providers, speech pathologists, endocrinologists, and other providers with expertise in hormone management that are trained to care for transgender patients. These providers can be available to work in a multidisciplinary approach with the aim to provide holistic healthcare for the transgender population and tailored to the individual needs of each patient.

## Methods

The objective of this paper is to describe the initiatives and programs developed and on-going to address the barriers to healthcare discussed above among the transgender population in a medically underserved, predominantly Hispanic urban area of the United States. We aim to explain the specific barriers to transgender healthcare in this unique binational region and propose interventions that will help the area to overcome these barriers. We hope that this paper will serve as a foundation or model for the development of a comprehensive transgender clinic in our community.

Although no official research was conducted, our findings are nevertheless a reflection of the experiences, challenges, and successes faced by PCPs, agencies, and researchers when trying to provide health interventions to the transgender population from a variety of disciplinary approaches. We found that it is very difficult for transgender individuals to obtain medical services on the U.S.–Mexico border, not only in terms of a culturally competent practice view, but also in terms of the severe shortage of medical expertise for transgender treatment on the primary- and specialty-care levels.

## Results

The following efforts were led by different teams to address the needs of the transgender community in El Paso, Texas including Safe Zone LGBT Allies Trainings; a transgender clinic with participation of primary care, endocrinology, gynecology, and mental health providers; a referral list of local LGBT-friendly healthcare and social services providers; and the transgender-specific voice modification clinic. Each of these efforts was led by a different team; however, all involved have been meeting since 2013 to collaborate and support each other's efforts.

### Safe Zone LGBT allies training

As a means to overcome a lack of knowledge, we took advantage of Safe Zone LGBT Allies trainings that are offered at the Texas Tech University Health Sciences Center (TTUHSC) El Paso campus, launched in 2014. Offering an Allies or Safe Zone program is among one of the first steps that an institution can take to achieve a community that embraces diversity and creates a learning environment that is accepting of LGBT individuals.^29^ These hour-long workshops, provided through the Office of Diversity, Inclusion, and Global Health, are available to all faculty, residents, staff, and medical and nursing students on campus. The trainings cover appropriate terminology used by the LGBT community, legislative policies, health disparities in the LGBT community, and tips on how to be an ally and provide culturally competent care.

Participants complete a pre- and post-workshop survey. Descriptive data from original survey administered in 2014–2016 (Table 1) and a revised survey administered in 2017 (Table 2) indicate that, overall, there was an increase in knowledge of LGBT identities and learning how to become a competent LGBT ally. Participants who completed the training received rainbow caduceus pins and Safe Zone placards to display in their work areas.^30^ To date, 304 faculty and residents, 171 medical and nursing students, and 94 staff on the TTUHSC El Paso campus have participated in these training workshops.

**Table 1.** **Descriptive Information from Safe Zone Training Pre- and Post-Workshop Surveys Among Faculty and Residents (*n*=224), Students (*n*=88), and Staff (*n*=86), 2014–2016**

	Faculty and residents		Students		Staff	
Survey items (2014–2016)	Pre-freq. (%) *n*=224	Post-freq. (%) *n*=141	Pre-freq. (%) *n*=88	Post-freq. (%) *n*=62	Pre-freq. (%) *n*=86	Post-freq. (%) *n*=57
*Please indicate your:* knowledge of LGBT identities						
Not at all	13 (5.8)	—	3 (3.4)	—	8 (9.3)	—
Minimally	37 (16.5)	3 (2.1)	11 (12.5)	—	19 (22.1)	4 (7.0)
No opinion	18 (8.0)	5 (3.6)	4 (4.5)	2 (3.3)	2 (2.3)	2 (3.5)
Somewhat	111 (49.6)	71 (50.4)	46 (52.3)	26 (41.9)	42 (48.8)	31 (54.4)
Substantially	34 (15.2)	59 (41.8)	24 (27.3)	34 (54.8)	9 (10.5)	20 (35.1)
Not applicable	—	1 (0.7)	—	—	1 (1.2)	—
Missing	11 (4.9)	2 (1.4)	—	—	5 (5.8)	—
Knowledge of LGBT-related institution climate issues						
Not at all	42 (18.8)	2 (1.4)	10 (11.3)	—	20 (23.3)	1 (1.8)
Minimally	71 (31.7)	10 (7.1)	29 (33.0)	3 (4.8)	24 (27.9)	15 (26.3)
No opinion	32 (14.3)	12 (8.5)	5 (5.7)	9 (14.6)	6 (7.0)	4 (7.0)
Somewhat	57 (25.4)	71 (50.4)	37 (42.0)	26 (41.9)	24 (27.9)	23 (40.3)
Substantially	11 (4.9)	43 (30.5)	7 (8.0)	23 (37.1)	3 (3.5)	14 (24.6)
Not applicable	—	2 (1.4)	—	1 (1.6)	4 (4.7)	—
Missing	11 (4.9)	1 (0.7)	—	—	5 (5.8)	—
Understanding of the role of an LGBT ally						
Not at all	48 (21.4)	2 (1.4)	7 (8.0)	—	17 (19.8)	2 (3.5)
Minimally	58 (25.9)	4 (2.9)	18 (20.4)	—	27 (31.4)	3 (5.3)
No opinion	20 (8.9)	9 (6.4)	12 (13.6)	3 (4.8)	8 (9.3)	1 (1.7)
Somewhat	68 (30.4)	65 (46.1)	32 (36.4)	24 (38.7)	23 (26.7)	20 (35.1)
Substantially	17 (7.6)	59 (41.8)	19 (21.6)	35 (56.5)	4 (4.7)	31 (54.4)
Not applicable	—	1 (0.7)	—	—	2 (2.3)	—
Missing	13 (5.8)	1 (0.7)	—	—	5 (5.8)	—
Competence to serve as an LGBT ally						
Not at all	51 (22.8)	4 (2.8)	10 (11.4)	1 (1.6)	21 (24.4)	1 (1.8)
Minimally	52 (23.2)	7 (5.0)	10 (11.4)	—	23 (26.8)	5 (8.7)
No opinion	27 (12.1)	9 (6.4)	16 (18.2)	3 (4.8)	11 (12.8)	9 (15.8)
Somewhat	57 (25.4)	66 (46.8)	30 (34.1)	22 (35.5)	18 (20.9)	19 (33.3)
Substantially	24 (10.7)	52 (36.9)	21 (23.9)	35 (56.5)	5 (5.8)	23 (40.4)
Not applicable	—	2 (1.4)	—	1 (1.6)	3 (3.5)	—
Missing	13 (5.8)	1 (0.7)	1 (1.0)	—	5 (5.8)	—

LGBT, lesbian, gay, bisexual, and transgender.

**Table 2.** **Descriptive Information from Safe Zone Training Pre- and Post-Workshop Surveys (*n*=88), 2017**

	Faculty, residents, and staff		Students	
Survey items (2017)	Pre-freq. (%) *n*=84	Post-freq. (%) *n*=88	Pre-freq. (%) *n*=83	Post-freq. (%) *n*=80
*I feel confident in explaining the following:* various LGBT identities				
Strongly disagree	6 (7.1)	19 (21.6)	18 (21.7)	40 (50.0)
Disagree	17 (20.3)	18 (20.5)	23 (27.7)	23 (28.7)
Neither agree nor disagree	27 (32.1)	9 (10.2)	22 (26.5)	2 (2.5)
Agree	17 (20.3)	17 (19.3)	8 (9.7)	6 (7.5)
Strongly agree	7 (8.3)	23 (26.1)	5 (6.0)	9 (11.3)
Missing	10 (11.9)	2 (2.3)	7 (8.4)	—
Legislative efforts and guidelines related to LGBT healthcare				
Strongly disagree	5 (6.0)	16 (18.2)	10 (12.1)	24 (30.0)
Disagree	15 (17.8)	18 (20.4)	21 (25.3)	31 (38.8)
Neither agree nor disagree	34 (40.5)	13 (14.8)	23 (27.7)	10 (12.5)
Agree	14 (16.7)	21 (23.9)	17 (20.5)	6 (7.5)
Strongly agree	6 (7.1)	18 (20.4)	5 (6.0)	8 (10.0)
Missing	10 (11.9)	2 (2.3)	7 (8.4)	1 (1.2)
Health disparities among the various LGBT groups				
Strongly disagree	5 (6.0)	18 (20.5)	16 (19.3)	39 (48.8)
Disagree	15 (17.9)	18 (20.5)	20 (24.1)	22 (27.5)
Neither agree nor disagree	28 (33.3)	9 (10.1)	23 (27.7)	2 (2.5)
Agree	20 (23.8)	21 (23.9)	7 (8.5)	4 (5.0)
Strongly agree	6 (7.1)	21 (23.9)	9 (10.8)	12 (15.0)
Missing	10 (11.9)	1 (1.1)	8 (9.6)	1 (1.2)
Reasons for not seeking healthcare among the LGBT population				
Strongly disagree	5 (6.0)	18 (20.5)	16 (19.3)	43 (53.7)
Disagree	14 (16.7)	19 (21.6)	22 (26.5)	20 (25.0)
Neither agree nor disagree	31 (36.9)	8 (9.1)	22 (26.5)	2 (2.5)
Agree	17 (20.2)	19 (21.6)	9 (10.9)	1 (1.3)
Strongly agree	7 (8.3)	23 (26.1)	7 (8.4)	14 (17.5)
Missing	10 (11.9)	1 (1.1)	7 (8.4)	—
Ways to increase LGBT sensitivity in my area or institution				
Strongly disagree	5 (6.0)	17 (19.3)	18 (21.7)	40 (50.0)
Disagree	14 (16.7)	20 (22.7)	23 (27.7)	21 (26.3)
Neither agree nor disagree	31 (36.9)	9 (10.2)	16 (19.3)	3 (3.7)
Agree	17 (20.2)	18 (20.5)	9 (10.9)	4 (5.0)
Strongly agree	7 (8.3)	21 (23.9)	10 (12.0)	11 (13.8)
Missing	10 (11.9)	3 (3.4)	7 (8.4)	1 (1.2)
Ways to enhance physician-patient interaction				
Strongly disagree	6 (7.1)	20 (22.7)	20 (24.1)	41 (51.2)
Disagree	15 (17.9)	17 (19.3)	22 (26.5)	22 (27.5)
Neither agree nor disagree	27 (32.1)	10 (11.4)	16 (19.3)	3 (3.8)
Agree	18 (21.5)	18 (20.5)	8 (9.7)	2 (2.5)
Strongly agree	8 (9.5)	22 (25.0)	9 (10.8)	12 (15.0)
Missing	10 (11.9)	1 (1.1)	8 (9.6)	—
Ways to be a competent LGBT ally				
Strongly disagree	6 (7.1)	21 (23.9)	19 (22.9)	45 (56.3)
Disagree	17 (20.3)	16 (18.2)	27 (32.6)	17 (21.2)
Neither agree nor disagree	28 (33.3)	11 (12.5)	14 (16.9)	2 (2.5)
Agree	16 (19.1)	16 (18.2)	8 (9.6)	3 (3.8)
Strongly agree	8 (9.5)	23 (26.1)	8 (9.6)	12 (15.0)
Missing	9 (10.7)	1 (1.1)	7 (8.4)	1 (1.2)

In 2017, departments were trained at one time; therefore, staff members were trained alongside faculty and residents.

To increase uniformity of exposure and knowledge, as it is not a mandatory training, the office has developed a clinic-based approach, offering training to all members of a particular healthcare team (e.g., nursing, staff, physicians) at one time, thus facilitating designation of the entire clinic, and not just specific providers, as competent to provide LGBT care. This approach was initiated in summer 2016, and to date we have trained 6 of the 14 clinical departments and all students, faculty, and staff from the School of Nursing.

Another crucial educational resource is the Gay Straight Campus Alliance (GaSCA) at TTUHSC El Paso, established in 2012. Multiple studies have demonstrated that the presence of gay–straight alliances serves to improve the institutional climate and decrease victimization of transgender individuals.^31^ Since its inception on our campus, GaSCA has organized quarterly campus-wide meetings to discuss issues relevant to LGBT healthcare. As part of these meetings, multiple guest speakers, including local community health workers, transgender patients from our region, and healthcare providers who care for transgender individuals, have shared their knowledge and experience with the campus community.

To address the knowledge gap among providers on campus as well as throughout our local community, staff members from the Office of Diversity, Inclusion, and Global Health along with representatives from GaSCA provided a presentation for faculty and staff for the El Paso Veterans Affairs Healthcare System in June 2016. That same month, the office also collaborated with a local Senator's office to provide open training in transgender healthcare to all local healthcare professionals.

In addition to the work done by the Office of Diversity, Inclusion, and Global Health, faculty from the transgender clinic have addressed the lack of knowledge among healthcare providers on a local as well as a national level. Faculty members regularly provide lectures on transgender healthcare to local university faculty and students, social workers, nurses, and PCPs throughout the El Paso community. Faculty also have presented at the 2015 National Latino AIDS Awareness Day Conference to discuss transgender healthcare and what is specifically being done in El Paso at the transgender clinic.

### Transgender clinic

Our transgender clinic opened at TTUHSC El Paso in July 2014 for the purpose of delivering transgender-specific healthcare to children, adolescents, and adults. El Paso, Texas, is a region well known for having poor access to healthcare. Another major barrier was the payer policy, as some individuals who seek transgender care are not covered by insurance. To overcome this barrier, a specific payer policy was instituted by the Department of Pediatrics that included tailored fees for payment by uninsured individuals, which assisted in overcoming the financial barrier to healthcare. Collaborations with the institution and private pharmacies were established to procure affordable prices for the medication necessary for cross-sex hormone therapy.

Mental health issues are common among the transgender population. Transgender individuals experience high rates of depression and suicidal ideation, particularly TransLatinas, as compared with the general population.^32–35^ According to current guidelines,^27,28^ eligibility and readiness criteria for transgender care include the participation of mental health providers in the diagnosis and treatment of comorbidities (e.g., depression, anxiety). Comprehensive healthcare and mental health services for transgender clients are a challenge in rural and urban areas.^36^ Often, patients come to our clinic self-referred or referred by other patients as opposed to mental health providers, making mental health another issue that needs to be addressed in the establishment of healthcare for the transgender population. To address this concern, we collaborated with the psychiatry department at our institution to bring prompt attention to the evaluation of transgender patients. Also, a clinical psychologist is part of the medical visit at the transgender clinic, for new patients and follow-ups. We also worked with mental health providers in the community outside of our academic institution to create a broader referral base for our patients. Staff in the transgender clinic are all bilingual, including most of the medical staff, for this reason, in particular, the language was not identified as a barrier to healthcare for the population served.

### LGBT-friendly healthcare and social services providers

Finding healthcare providers who are willing to participate is another concern related to providing healthcare to transgender patients in our region. One barrier identified by transgender women in the TWIST Study was a lack of knowledge of resources for this population in El Paso.^10^ To reduce barriers to healthcare and social services in the transgender community, local efforts have led to the first version of a referral list of self-identified LGBT-competent providers, including providers who are transgender friendly: “The Purple Pages of El Paso.”^37^ This list is intended to be used by members of the LGBT community who seek services as well as by providers who need to refer their patients to other services. The referral list is hosted by the City of El Paso Department of Public Health website. The efforts to identify transgender-competent providers in our area have led to the formation of a multidisciplinary healthcare group that includes endocrinologists to provide adequate transgender hormonal therapy according to current guidelines; mental health providers, including psychiatrists, psychologists, counselors and social workers; fertility specialists who are willing to provide services for sperm preservation before cross-hormone therapy; and gynecologists to provide necessary primary care for female-to-male transgender patients.

Collaboration with other institutions in our community has led to the development of a transgender-specific voice-training program. The program encompasses all areas of communication including pitch, resonance, prosody, and articulation as well as gender-specific, non-verbal language, and social rules of communication.^38–40^

### Voice modification clinic

The Voice Modification Clinic for Transgender Individuals at The University of Texas El Paso (UTEP) was developed in response to a transwoman's parent's call in search of voice services for her daughter. The parent reported that she had called several speech therapy clinics in the area with no success. Further, she reported feelings of frustration in regard to herself and, more importantly, for her daughter.

Up to that point, voice feminization and masculinization were covered in course material provided to the graduate students in the UTEP Speech-Language Pathology Program (SLPP). Nevertheless, the students did not have the opportunity to develop the clinical skills needed for this communication parameter, given that our on-site speech, language, and hearing clinic did not have any transgender individuals in the caseload. Therefore, we agreed to provide voice feminization treatment to this individual.

During the initial evaluation session, the patient reported that she was a patient of the transgender clinic at the academic center. The faculty in SLPP decided to contact the transgender clinic and offer voice modification services at no cost to thier transgender patients. This referral system serves a dual purpose: (1) to provide a service to the community at no cost and (2) to provide graduate student clinicians in the SLPP with the opportunity to gain clinical experience with this communication parameter.

In the last 3 years, the Voice Modification Clinic has provided treatment to 4 transgender men and 14 transgender women. Four of the patients have been discharged due to meeting their treatment goals. During this same time frame, 57 students have completed SLPP, and all participated in Safe Zone training provided by TTUHSC El Paso. Our referrals now come not only from the physicians but also from the community, including transgender support groups, friends, family members, and mental health providers. Recently, we have had self-referrals, as these individuals have heard about our services from The Purple Pages of El Paso^37^ and via word of mouth. The increase in our referrals suggests that we are providing a service that is much needed in our underserved community. Further, this model has provided valuable experience and training on voice modification intervention to future speech-language pathologists.

## Conclusion

Transgender patients suffer from a lack of accessible healthcare and competent providers to deliver this care. Several barriers related to communication have been identified in transgender individuals in this MUA, including cultural, linguistic, and low literacy issues. This problem is often magnified for Hispanics and on the U.S.–Mexico border due to culture-specific barriers and the baseline shortage of medical providers in this underserved area. Identification of the specific barriers and the appropriate providers as well as the means to overcome these barriers is extremely important—once identified, a specific plan can be established. A multidisciplinary approach is crucial for the development of successful programs for transgender healthcare. Our community is in great need of a well-established clinic that can provide multidisciplinary care for this population and for which this review is intended to serve as a reference. Some important aspects for the development of successful programs are to determine and address the medical knowledge gap and biases of medical students, physicians in training, attending physicians, educators, and support staff. The revision of clinic intake forms is also crucial to creating an inclusive environment for transgender patients and should help to mitigate the initial fear that transgender individuals face when accessing healthcare. The Office of Diversity, Inclusion, and Global Health is currently working with the electronic medical records system administrators at TTUHSC El Paso to include two additional fields: preferred name and preferred pronoun.

Although research was not conducted or presented in this paper, we recognize that research priorities include identifying the overall ability to deliver care to transgender patients as well as assessing barriers related to ethnic/racial status or geographic location. Additional efforts should focus on addressing the financial gaps common in impoverished areas as well as developing mechanisms of cultural validation for research instruments. More research is needed to determine how to overcome such barriers as societal stigma and to address the mental health of the families of these patients. Evidence indicates a high prevalence of psychiatric problems diagnosed in this community, and, thus, there is a need for longitudinal studies to understand the effect of risk factors and optimal timing for psychosocial interventions.^41^

Research also should address the specific risk factors of transgender individuals who belong to a minority ethnic group and/or live in poverty. This calls for the need to modify or create valid screening instruments that allow us to better understand the needs of transgender individuals. Research also should focus on transgender health beyond transitioning, including how this process affects health, well-being, and quality of life as a means to promote successful aging and full incorporation into society.^42^

Further research conducted in our community will help to highlight the magnitude of the need for transgender healthcare. Having provided a needs assessment of and the foundation for comprehensive transgender healthcare, the creation of a transgender clinic should be justified and supported. Our ultimate goal is to create a space to provide an integrated and multidisciplinary health service for the transgender community, in which treatment is individualized and carried out by health providers trained in transgender healthcare within their particular specialty. Our expected outcome is to unify and support transgender individuals, to create sensitivity and awareness in our community, and to overcome the barriers that we have encountered when providing health services. In the future, we plan to establish a stronger, sustainable collaborative to better serve the transgender community in the region and to serve as a model for other low-resource settings.

## Abbreviations Used

GaSCA: Gay Straight Campus Alliance
HRSA: Health Resources and Services Administration
MSM: men who have sex with men
MUA: medically underserved area
PCP: primary care physicians
SLPP: Speech-Language Pathology Program
TTUHSC: Texas Tech University Health Sciences Center
TWIST: Transgender Women's Identity, Sexuality, and Transition
